# Phenotypic characteristics of the p.Asn215Ser (p.N215S) *GLA* mutation in male and female patients with Fabry disease: A multicenter Fabry Registry study

**DOI:** 10.1002/mgg3.389

**Published:** 2018-04-12

**Authors:** Dominique P. Germain, Eva Brand, Alessandro Burlina, Franco Cecchi, Scott C. Garman, Judy Kempf, Dawn A. Laney, Aleš Linhart, László Maródi, Kathy Nicholls, Alberto Ortiz, Federico Pieruzzi, Suma P. Shankar, Stephen Waldek, Christoph Wanner, Ana Jovanovic

**Affiliations:** ^1^ Division of Medical Genetics University of Versailles Paris‐Saclay University Montigny France; ^2^ Department of Nephrology, Hypertension and Rheumatology University Hospital Münster Münster Germany; ^3^ Neurological Unit St Bassiano Hospital Bassano del Grappa Italy; ^4^ Referral Center for Cardiomyopathies Cardiothoraco‐vascular Department Careggi University Hospital Florence Italy; ^5^ Department of Biochemistry and Molecular Biology University of Massachusetts Amherst Amherst MA USA; ^6^ Formerly Sanofi Genzyme Cambridge MA USA; ^7^ Department of Human Genetics Emory University School of Medicine Atlanta GA USA; ^8^ Second Department of Medicine ‐ Department of Cardiovascular Medicine 1st Faculty of Medicine Charles University Prague Czech Republic; ^9^ Department of Infectious and Pediatric Immunology University of Debrecen Debrecen Hungary; ^10^ Department of Nephrology Royal Melbourne Hospital, and Department of Medicine University of Melbourne Parkville VIC Australia; ^11^ Unidad de Dialisis IIS‐Fundación Jiménez Díaz School of Medicine UAM, IRSIN and REDINREN Madrid Spain; ^12^ Department of Medicine and Surgery Nephrology Unit University of Milano‐Bicocca Monza Italy; ^13^ Department of Pediatrics Division of Genomic Medicine UC Davis School of Medicine Sacramento CA USA; ^14^ University of Sunderland Sunderland UK; ^15^ Renal Division University Hospital of Würzburg Würzburg Germany; ^16^ Mark Holland Metabolic Unit Salford Royal NHS Foundation Trust Salford UK

**Keywords:** cardiac variant, Fabry disease, *GLA*, p.Asn215Ser, p.N215S, phenotype

## Abstract

**Background:**

The p.Asn215Ser or p.N215S *GLA* variant has been associated with late‐onset cardiac variant of Fabry disease.

**Methods:**

To expand on the scarce phenotype data, we analyzed natural history data from 125 p.N215S patients (66 females, 59 males) enrolled in the Fabry Registry (NCT00196742) and compared it with data from 401 patients (237 females, 164 males) harboring mutations associated with classic Fabry disease. We evaluated interventricular septum thickness (IVST), left ventricular posterior wall thickness (LVPWT), estimated glomerular filtration rate and severe clinical events.

**Results:**

In p.N215S males, mildly abnormal mean IVST and LVPWT values were observed in patients aged 25–34 years, and values gradually increased with advancing age. Mean values were similar to those of classic males. In p.N215S females, these abnormalities occurred primarily in patients aged 55–64 years. Severe clinical events in p.N215S patients were mainly cardiac (males 31%, females 8%) while renal and cerebrovascular events were rare. Renal impairment occurred in 17% of p.N215S males (mostly in patients aged 65–74 years), and rarely in females (3%).

**Conclusion:**

p.N215S is a disease‐causing mutation with severe clinical manifestations found primarily in the heart. Cardiac involvement may become as severe as in classic Fabry patients, especially in males.

## 1 INTRODUCTION

Fabry disease (OMIM #301500) is an X‐linked lysosomal storage disorder caused by mutations in the *GLA* (OMIM #300644; HGNC 4296; NCBI reference sequence NM_000169.2) gene encoding enzyme α‐galactosidase (α‐Gal, EC 3.2.1.22; Uniprot P06280) (Desnick, Ioannou, & Eng, 2001; Germain, 2010). Patients with the classic, severe form of Fabry disease have severely reduced or absent α‐Gal activity leading to progressive lysosomal substrate accumulation [globotriaosylceramide (GL‐3) in particular] in plasma and in a variety of cell types, including vascular endothelial and smooth muscle cells, podocytes and other kidney cell types and cardiomyocytes (Germain, 2010). In classically affected male patients, onset of clinical symptoms occurs in pediatric patients (Hopkin et al., 2008; Laney et al., 2015; Ramaswami et al., 2006), and life‐threatening complications involving the kidneys, heart and cerebrovascular system may develop with advancing age (Germain, 2010; Germain et al., 2013; Ortiz et al., 2008, 2010; Patel et al., 2011; Senechal & Germain, 2003; Sims, Politei, Banikazemi, & Lee, 2009; Wilcox et al., 2008). Cardiac events are the most frequently reported cause of death in the overall Fabry patient population (Mehta et al., 2009; Waldek, Patel, Banikazemi, Lemay, & Lee, 2009). In heterozygotes, clinical presentations are more variable than in male patients and are dependent on multiple factors including the type of mutation and the X‐chromosome inactivation profile (Echevarria et al., 2016; Germain, 2010; Wilcox et al., 2008).

To date, several predominantly missense mutations have been reported to be associated with a later‐onset cardiac phenotype of Fabry disease, including p.N215S, p.M296I, p.R301Q, p.G328R and IVS4+919G>A (Eng, Resnick‐Silverman, Niehaus, Astrin, & Desnick, 1993; Eng et al., 1994; Germain, 2001, 2010; Germain et al., 1996; Ishii, Kase, Sakuraba, & Suzuki, 1993; Ishii, Nakao, Minamikawa‐Tachino, Desnick, & Fan, 2002; Ishii, Sakuraba, & Suzuki, 1992; Ishii et al., 2007; Nakao et al., 1995; Sachdev et al., 2002; Sakuraba et al., 1990; von Scheidt et al., 1991; Spada et al., 2006; Yoshitama et al., 2001). Of these mutations, p.N215S appears to be the most common mutation in Western countries (personal communication). It has been reported that cardiac variant patients may have significant levels of residual α‐Gal activity [2%–20% of normal (Germain, 2001; Ishii et al., 1993, 2007; Spada et al., 2006)] and generally lack the early classic symptoms of Fabry disease (e.g., neuropathic pain, gastrointestinal complaints, angiokeratoma, cornea verticillata), with manifestations being confined to the heart predominantly (left ventricular hypertrophy, conduction disturbances, arrhythmias). These patients generally present later in life than classic patients (Elleder et al., 1990; Germain, 2010; Nakao et al., 1995; Patel et al., 2015; Sachdev et al., 2002; von Scheidt et al., 1991), or may be diagnosed earlier as a result of neonatal, family or high‐risk population screening initiatives.

The p.Asn215Ser missense mutation [nomenclature recommended by the Human Genome Variation Society (den Dunnen et al., 2016); usually referred to as p.N215S] results from an A‐to‐G transition at codon 215 in exon 5 of *GLA* (c.644A>G), with subsequent substitution of a glycosylated asparagine by a serine. Glycosylation at this site is crucial for the formation of soluble, active enzyme that can be transported to the lysosome (Ioannou, Zeidner, Grace, & Desnick, 1998).

The purpose of our study was to determine the phenotypic characteristics of patients with the Fabry p.N215S mutation, and to compare them with the characteristics of patients harboring mutations associated with classic Fabry disease followed at the same clinical centers providing specialized care to the p.N215S patients.

## 2 PATIENTS AND METHODS

### 2.1 Patients

This study includes male and female patients enrolled in the Fabry Registry (age range: 0.5–72.5 years at first assessment) diagnosed with Fabry disease and harboring the p.N215S mutation. All patients were from 10 international Fabry Registry sites that had contributed clinical data for a minimum of six p.N215S patients, and had received specialized care supervised by one of the authors (D.P.G., E.B., F.C., S.P.S./D.A.L., A.L., L.M., K.N., F.P., S.P.S., C.W., A.J.).

### 2.2 Outcomes

We analyzed natural history data (i.e., obtained before initiation of enzyme replacement therapy [ERT], if indicated) including left ventricular posterior wall thickness (LVPWT), interventricular septum thickness (IVST) and estimated glomerular filtration rate (eGFR) (bedside Schwartz equation if <18 years of age, Chronic Kidney Disease Epidemiology Collaboration [CKD‐EPI] equation if ≥18 years of age). The first values entered in the Fabry Registry were used for analysis. We also evaluated severe clinical events including: (1) Cardiac events: angina pectoris, arrhythmia, congestive heart failure, myocardial infarction, implantation of cardiac devices, significant cardiac procedure; (2) Renal events: first occurrence of either the initiation of chronic dialysis (>40 days) or renal transplantation; (3) Cerebrovascular events: hemorrhagic or ischemic stroke; and (4) Death. The first severe clinical event that occurred during natural history follow‐up and time to first clinical event was analyzed for each of the event types. We analyzed electrocardiogram (ECG) data separately to further define arrhythmia subtypes. We compared clinical data from p.N215S patients to Fabry Registry data from patients harboring *GLA* mutations associated with classic Fabry phenotype followed at the same clinical centers. The mutation classification “classic Fabry disease” for these patients was verified using data from the Fabry‐database.org mutation database (Fabry disease mutation database, [Link]). The final list of classic mutations (Table S1) was reviewed and approved by a senior geneticist experienced in the molecular diagnosis and management of Fabry patients (D.P.G).

### 2.3 Statistical methods

We analyzed IVST, LVPWT and eGFR data by age group: <25 years; 25–34 years; 35–44 years; 45–54 years; 55–64 years; and 65–74 years. Kaplan–Meier models depicting estimates of time to first reported event during natural history follow‐up were performed for p.N215S and classic Fabry patients of both genders. We used descriptive statistics to characterize the individuals within the respective age groups. Statistical analyses were performed using SAS statistical software V.9.2 (SAS Institute Inc., Cary, NC, USA).

### 2.4 Fabry Registry

The Fabry Registry (NCT00196742; sponsor: Sanofi Genzyme) is a multicenter, international, longitudinal, observational program designed to track the natural history and outcomes of patients with Fabry disease (Fabry Registry website, [Link]). Patient and investigator participation is voluntary. Each independent site is responsible for obtaining patients’ informed written consent to submit their health information to the Registry, and to use and disclose this information in aggregate analyses. The Registry protocol, informed consent form and any locally required authorization documents to send patient information to the Registry are reviewed and approved by the local fully constituted Institutional Review Board (IRB) or Independent Ethics Committee (IEC) unless the site provides the Registry with documentation that approval is not required or has been waived by a particular IRB/IEC.

## 3 RESULTS

### 3.1 Patient demographics

Data from 125 patients (59 males, 66 females) with the p.N215S genotype from the 10 centers were available in the Fabry Registry; the total number of p.N215S patients enrolled worldwide was 231 (105 males, 126 females). Mean age (range) at first assessment of IVST, LVPWT and eGFR in p.N215S males was 49.1 (8.7–72) years, 50.6 (8.7–71.4) years and 51.9 (8.7–72.5) years, respectively. For p.N215S females, mean age (range) was 41 (0.5–69.3) years for IVST and LVPWT, and 41 (5.8–69.2) years for eGFR. These data were compared to data from 401 patients (164 males, 237 females) harboring classic Fabry disease mutations followed at the same centers.

### 3.2 IVST and LVPWT

**Figure 1:**
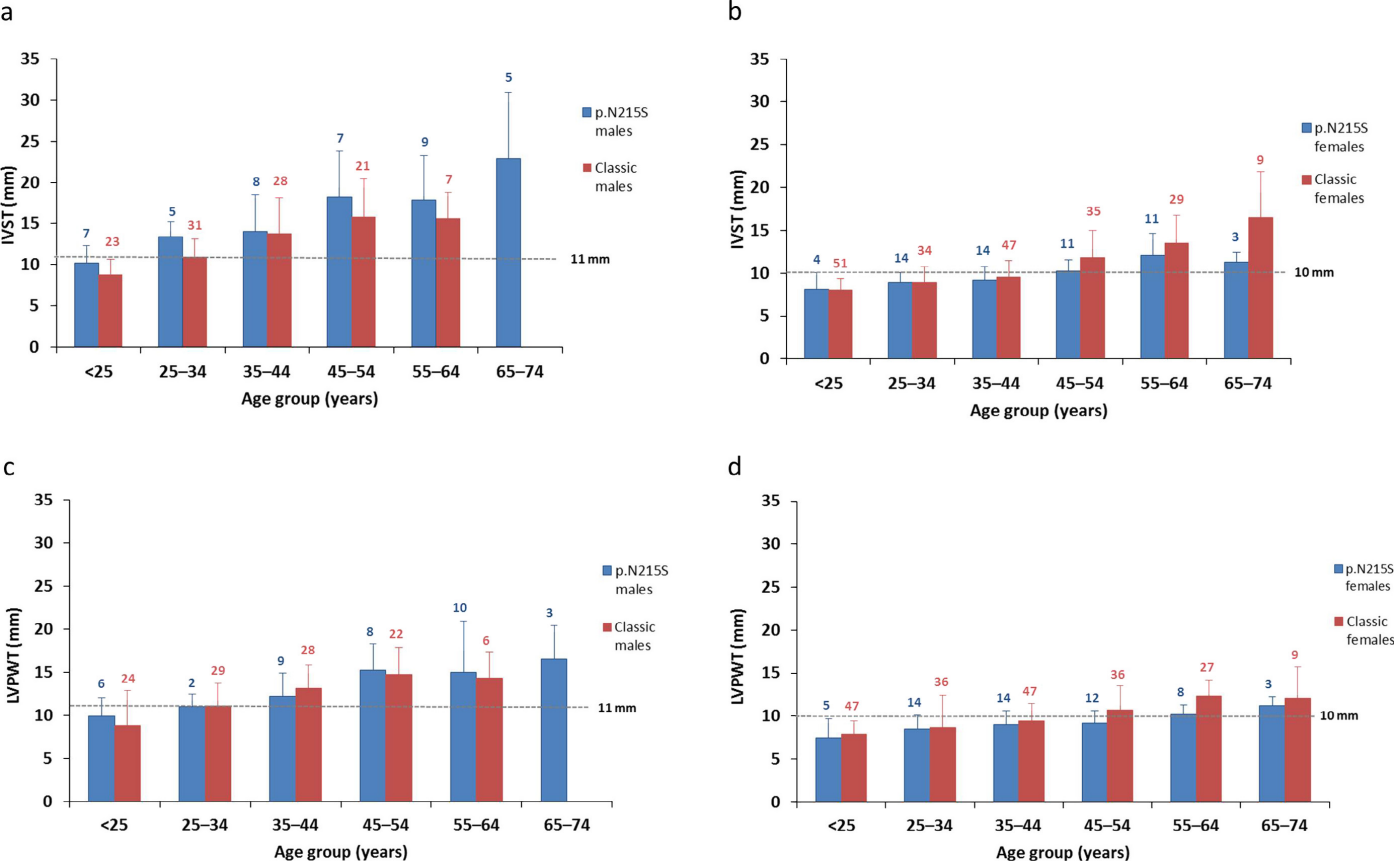
Interventricular septum thickness (IVST) and left ventricular posterior wall thickness (LVPWT) values at first assessment during natural history follow‐up. IVST values are shown for male (a) and female (b) p.N215S (blue) and classic (red) patients. LVPWT data are shown for male (c) and female (d) p.N215S (blue) and classic (red) patients. Ranges in males: normal, 6–10 mm; mildly abnormal, 11–13 mm; moderately abnormal, 14–16 mm; severely abnormal, >16 mm. Ranges in females: normal, 6–9 mm; mildly abnormal, 10–12 mm; moderately abnormal, 13–15 mm; severely abnormal, >15 mm (Lang et al., 2015). Data are presented as mean and *SD*

For p.N215S males, overall, 88% and 74% had an abnormal value [≥11 mm (Lang et al., 2015)] for IVST and LVPWT, respectively, at first assessment during natural history follow‐up. Mean (standard deviation [*SD*]) IVST and LVPWT values were first mildly abnormal in the 25–34‐year age group with values of 13.4 (1.8) mm (Figure 1a) and 11.0 (1.4) mm (Figure 1c), respectively; mean values gradually increased with advancing age to severely abnormal mean values of 22.9 (8.0) (Figure 1a) and 16.5 (3.9) mm (Figure 1c), respectively, in the 65–74‐year age group.

For classic male patients, mean (*SD*) IVST and LVPWT values were also first mildly abnormal for the 25–34‐year age group, with values of 11.0 (2.2) mm (Figure 1a) and 11.1 (2.6) mm (Figure 1c), respectively; values increased with advancing age to maximum moderately abnormal mean values of 15.8 (4.6) mm (Figure 1a) and 14.7 (3.1) mm (Figure 1c), respectively, in the 45–54‐year age group. No classic male patients reported their first natural history assessment at age 65–74 years.

For p.N215S females, overall, 49% and 34% of the females had an abnormal value [≥10 mm (Lang et al., 2015)] for IVST and LVPWT, respectively, at first assessment. Mean (*SD*) IVST was first mildly abnormal in the 45–54‐year age group [10.2 (1.3) mm (Figure 1b)] and mean (*SD*) LVPWT was mildly abnormal in the 55–64‐year age group [10.2 (1.1) mm (Figure 1d)], and remained mildly abnormal with advancing age.

For classic female patients, IVST and LVPWT were abnormal from age 45–54 years onward (Figure 1b,d). IVST and LVPWT reached a maximum mean (*SD*) value in patients aged 65–74 years [16.5 (5.6) mm (severely abnormal)] and 55–64 years [12.3 (1.9) mm (mildly abnormal)], respectively.

### 3.3 eGFR

**Figure 2:**
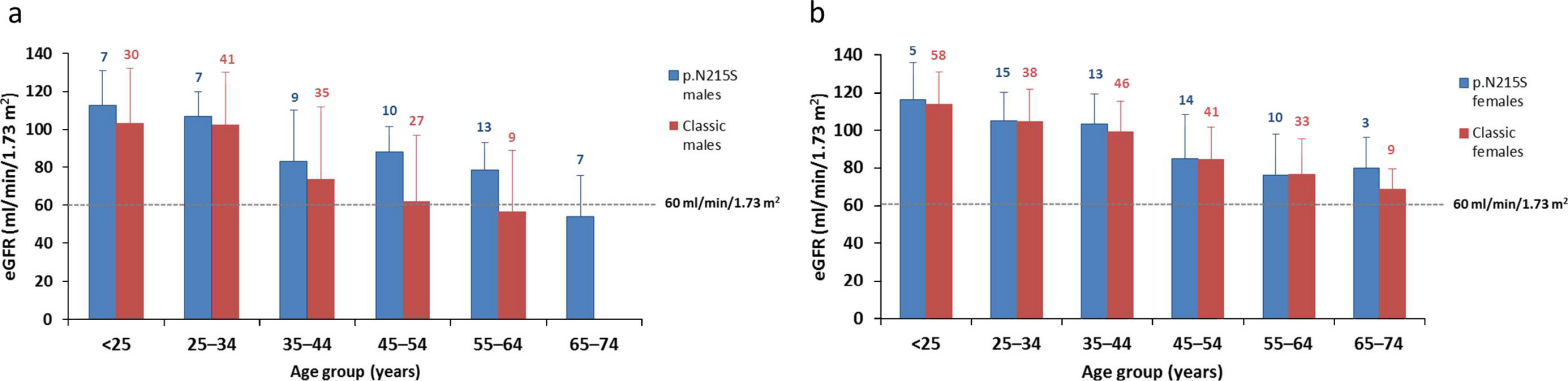
Estimated glomerular filtration rate (eGFR) values at first assessment during natural history follow‐up. eGFR values are shown for male (a) and female (b) p.N215S (blue) and classic (red) patients. Values <60 ml/min/1.73 m^2^ indicate the presence of chronic kidney disease. Data are presented as mean and *SD*

For p.N215S patients, overall, 17% of males and 3% of females had an eGFR value at first assessment during natural history follow‐up of <60 ml/min/1.73 m^2^, suggestive of chronic kidney disease. For both genders, p.N215S patients’ mean eGFR values remained >60 ml/min/1.73 m^2^ for all age groups except males aged 65–74 years [mean eGFR (*SD*) 54.1 (21.5) ml/min/1.73 m^2^] (Figure 2a).

For classic male patients, mean eGFR was first abnormal in the 55–64‐year age group [mean eGFR (*SD*) 56.8 (32.0) ml/min/1.73 m^2^; there were no males with first assessments at age 65–74 years], whereas for classic females, mean eGFR values were above the lower limit of normal in all age groups (Figure 2b).

### 3.4 Severe clinical events

First severe clinical events in p.N215S males (32%) occurred at a mean age of 52.4 years, approximately 14 years later as compared to classic male patients (33%), whereas in p.N215S females (9%) and classic female patients (20%) first events occurred at a similar mean age (approximately 50 years) (Table 1).

**Table 1:** Severe clinical events reported as first clinical event in p.N215S and classic Fabry disease patients from the clinical centers participating in the study

Severe clinical events	p.N215S males (*n *=* *59)		Classic males (*n *=* *164)		p.N215S females (*n *=* *66)		Classic females (*n *=* *237)	
	*n* (%)	Mean age (*SD*), years	*n* (%)	Mean age (*SD*), years	*n* (%)	Mean age (*SD*), years	*n* (%)	Mean age (*SD*), years
Any event	19 (32)	52.4 (11.8)	54 (33)	38.3 (10.9)	6 (9)	50.2 (11.6)	47 (20)	49.2 (13.6)
Cardiac event	18 (31)	52.3 (12.1)	34 (21)	43.3 (10.6)	5 (8)	51.1 (12.8)	36 (15)	50.2 (13.6)
Angina pectoris	‐	‐	9 (5)	‐	3 (5)	‐	14 (6)	‐
Arrhythmia	10 (17)	‐	13 (8)	‐	1 (2)	‐	14 (6)	‐
Congestive heart failure	1 (2)	‐	4 (2)	‐	‐	‐	‐	‐
Myocardial infarction	1 (2)	‐	2 (1)	‐	‐	‐	‐	‐
Significant cardiac procedure	6 (10)	‐	6 (4)	‐	1 (2)	‐	8 (3)	‐
Renal event	‐	‐	25 (15)	36.9 (11.3)	1 (2)	45.0	1 (<1)	41.0
Chronic dialysis	‐	‐	23 (14)	‐	‐	‐	‐	‐
Transplant	‐	‐	2 (1)	‐	1 (2)	‐	1 (<1)	‐
Cerebrovascular event	1 (2)	54.1	12 (7)	34.8 (8.8)	‐	‐	15 (6)	47.2 (13.7)
Hemorrhagic stroke	‐	‐	2 (1)	‐	‐	‐	‐	‐
Ischemic stroke	1 (2)	‐	10 (6)	‐	‐	‐	15 (6)	‐
Death	‐	‐	2 (1)	47.0 (7.7)	‐	‐	1 (<1)	66

SD, standard deviation.

p.N215S males primarily had cardiac events (31%), with arrhythmia and significant cardiac procedure being most prevalent. No p.N215S males reported angina pectoris whereas 5% of p.N215S females reported this type of event; in total, 8% of p.N215S females reported a cardiac event (Table 1).

Only one male p.N215S patient had a cerebrovascular event (ischemic stroke, age 54 years, no atrial fibrillation), and only one female p.N215S patient had a renal event (dialysis, age 45 years) as first severe event (Table 1). The analysis of severe events included those events that occurred as first event. It should be noted that four p.N215S patients experienced a renal event not documented as first severe event during natural history follow‐up (i.e., kidney transplant in one 54‐year‐old male; dialysis in one 39‐year‐old male, followed by kidney transplant at age 43 years; and dialysis in two females aged 51 and 63 years) for a total of 5/125 (4%) renal events as first event or during follow‐up, all before age 65 years.

Cardiac events in classic males were less frequent as compared to p.N215S males (21% vs. 31%) (Table 1), with arrhythmia and angina pectoris being most common. Cardiac events occurred at younger ages in classic males compared to p.N215S males (mean age 43 years vs. 52 years). Mean age of cardiac events was similar for classic females and p.N215S females (approximately 50 years).

Substantially more noncardiac first events occurred in classic males compared to p.N215S males [i.e., renal events (15%) and cerebrovascular events (7%), with ischemic stroke being most prevalent (6%)]. Ischemic stroke occurred in 6% of classic females, but did not occur in p.N215S females (Table 1).

No deaths were reported in p.N215S patients as first severe clinical event, whereas three classic patients died (Table 1).

Separate analysis of arrhythmia data from ECGs showed that p.N215S males primarily had sinus bradycardia (7%), atrial fibrillation (4%) and other rhythm abnormalities (4%). Sinus bradycardia was the most prevalent type of arrhythmia in p.N215S females (7%). Arrhythmia subtypes in classic patients did not substantially differ as compared to p.N215S patients.

**Figure 3:**
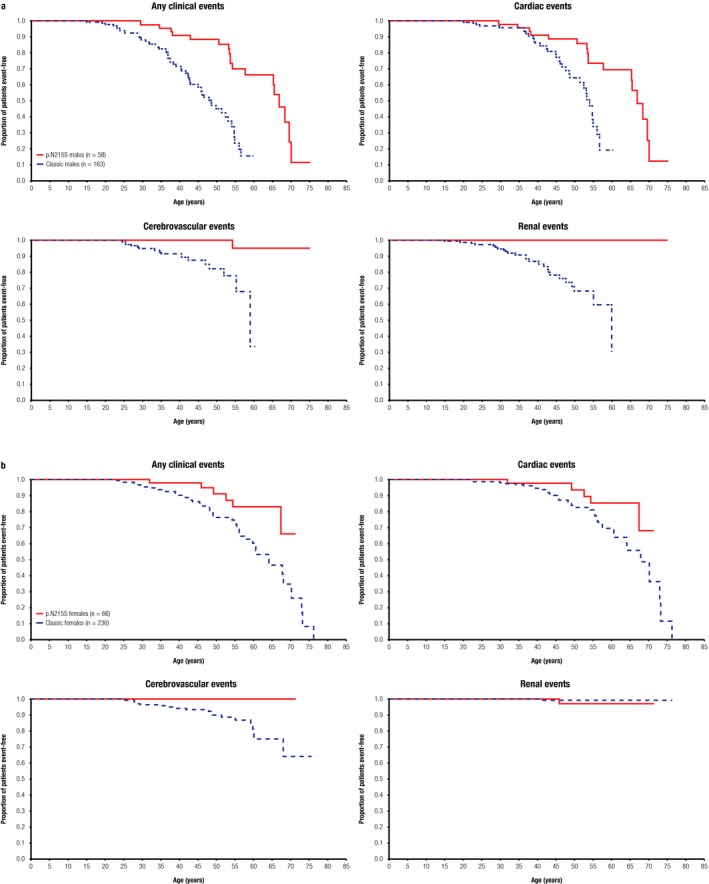
Kaplan–Meier curves depicting estimates of time to first severe clinical event. Data are shown for any clinical events, cardiac events, cerebrovascular events and renal events in male p.N215S (solid line) and classic (dashed blue line) patients (a); the same data are presented for female patients (b). Top left panels indicate any clinical events, top right panels cardiac events, bottom left panels cerebrovascular events, and bottom right panels renal events

Kaplan–Meier models depicting estimates of time to first reported severe clinical event (any, cardiac, cerebrovascular, renal) during natural history follow‐up are shown for p.N215S and classic patients of both genders (Figure 3a,b). For male p.N215S patients, cardiac events started to occur in the fourth decade of life, similar to classic males, whereas in female p.N215S patients, cardiac events were less common and appeared to first occur in the sixth decade of life.

### 3.5 Patients initiating ERT

Among p.N215S patients, 76% of males and 21% of females were initiated on ERT, at a mean age (*SD*) of 49.1 (15.7) years and 43.6 (14.2) years, respectively. Among classic patients, 92% of males and 46% of females were initiated on ERT, at a mean age (*SD*) of 36.6 (13.1) years and 46.3 (13.0) years, respectively.

## 4 DISCUSSION

The p.N215S missense mutation has been labeled as a later‐onset cardiac phenotype of Fabry disease (Eng et al., 1993, 1994; Germain, 2010; Germain et al., 1996; Ishii et al., 1992, 2002; Nakao et al., 1995; Patel et al., 2015; von Scheidt et al., 1991); however, published phenotypic data are still scarce. Three publications reported clinical findings in cohorts of patients with this mutation, but lacked detailed gender‐specific clinical information (Arends et al., 2017; Havranek, Linhart, Urbanova, & Ramaswami, 2013; Patel et al., 2015). In this Fabry Registry study, we analyzed data from clinical descriptions of 59 male and 66 female p.N215S patients, making it the largest cohort reported to date.

Our study confirms that p.N215S is a disease‐causing Fabry mutation with severe clinical manifestations essentially limited to the heart until late adulthood, especially in males. The majority of p.N215S males had mildly abnormal IVST and LVPWT values at first assessment, progressing, from age 25–34 years onward, to severely abnormal mean values in elderly patients, and almost all first severe clinical events reported were cardiac (31% of patients). A minority of p.N215S males had a slightly abnormal eGFR value at first assessment, most frequently among those aged 65–74 years. Renal and cerebrovascular events reported as first events were extremely rare. In p.N215S heterozygotes, IVST, LVPWT and eGFR abnormalities were rare across all age groups with exception of IVST, which was abnormal in patients aged 55–64 years. Cardiac events occurred in 8% of female p.N215S patients. No deaths were reported during natural history follow‐up in the p.N215S population.

Mean IVST and LVPWT values in p.N215S patients were not distinctly different from those in patients harboring mutations associated with classic Fabry disease with the exception that classic females in the oldest age groups seemed more affected – no classic males had first natural history assessments at age 65–74 years. The frequency of cardiac first events was higher in p.N215S males than in classic males (31% vs. 21%); however, events occurred at a later age (52.3 vs. 43.3 years). The higher rate of renal events in classic males (15%) may have contributed to the earlier occurrence of cardiac events given the well‐established association between renal failure and greater cardiovascular risk (Ortiz et al., 2014). Cardiac events were less frequent in p.N215S females than in classic females (8% vs. 15%) but occurred at a similar mean age. Tissue biopsies or autopsy studies of cardiac phenotypes of Fabry disease have revealed GL‐3 accumulation in cardiomyocytes but not in the vasculature of the heart, or other tissues (Desnick et al., 2001; Elleder et al., 1990; von Scheidt et al., 1991), suggesting that residual levels of α‐Gal can limit GL‐3 accumulation in the endothelium and, theoretically, the risk of Fabry‐related angina pectoris or myocardial infarction (Mayes, Cray, Dell, Scheerer, & Sifers, 1982).

In the current study, 17% of p.N215S males (primarily patients aged 65–74 years) had an abnormal eGFR value at first assessment; mean eGFR in classic males was abnormal a decade earlier. No renal events were reported as first event for male p.N215S patients, and these events occurred very rarely during natural history follow‐up in all female patients. In total, five patients (three females) required renal replacement therapy during the course of their disease (not reported as first severe event for four patients). This illustrates that, although the p.N215S variant is primarily cardiac in nature, a minority of p.N215S patients may develop renal impairment later in life, which may or may not be secondary to cardiac compromise. In addition, in patients with kidney function abnormalities, exclusion of non‐Fabry‐related medical conditions causing kidney disease (e.g., diabetes, heart failure, hypertension, primary glomerulonephritis, collagen polymorphisms) as well as identification of risk factors, such as obesity, hypercholesterolemia, smoking habits, alcohol consumption, nephrotoxic drugs (i.e., anticoagulants, diuretics) and contrast media, is warranted. Moreover, renal function declines with age in the normal adult population; the reported rate of loss of eGFR, beginning at age 40 years, is 1 ml/min/1.73 m^2^/year (Lindeman, Tobin, & Shock, 1985). GFR and albuminuria/proteinuria should be monitored regularly once an abnormality has been detected. Regarding the possibility of Fabry‐related primary kidney disease, a single case of a 75‐year‐old p.N215S male with proteinuria, mildly decreased renal function attributed to arteriosclerosis, and GL‐3 accumulation mostly limited to podocytes (absent from most other renal cell types, including vascular endothelium) has been reported to date (Meehan, Junsanto, Rydel, & Desnick, 2004).

The observation that cardiac involvement in p.N215S patients can become as severe as, and sometimes even more severe than, in patients with classic Fabry mutations may be due to silent progression of cardiac disease until the (often delayed) diagnosis and subsequent enrollment in the Fabry Registry. Characteristic early‐onset Fabry symptoms observed in classic patients (e.g., neuropathic pain, angiokeratoma, cornea verticillata) are reportedly rare in p.N215S patients, but may occur (Arends et al., 2017). Thus, early symptoms generally cannot alert the clinician to a possible diagnosis of Fabry disease and trigger therapeutic intervention. Fabry disease screening, through evaluation of α‐Gal activity in plasma/leukocytes and genetic analysis in individuals with hypertrophic cardiomyopathy aged ≥30 years, may identify these patients. However, the presence of a sarcomeric or a transthyretin mutation should also be ruled out.

Stroke as first severe clinical event occurred in only one male p.N215S patient without evidence of atrial fibrillation on ECGs. To rule out silent episodic atrial fibrillation as cardioembolic cause of stroke, ECG‐Holter prolonged monitoring or loop recorders may be useful tools.

**Figure 4:**
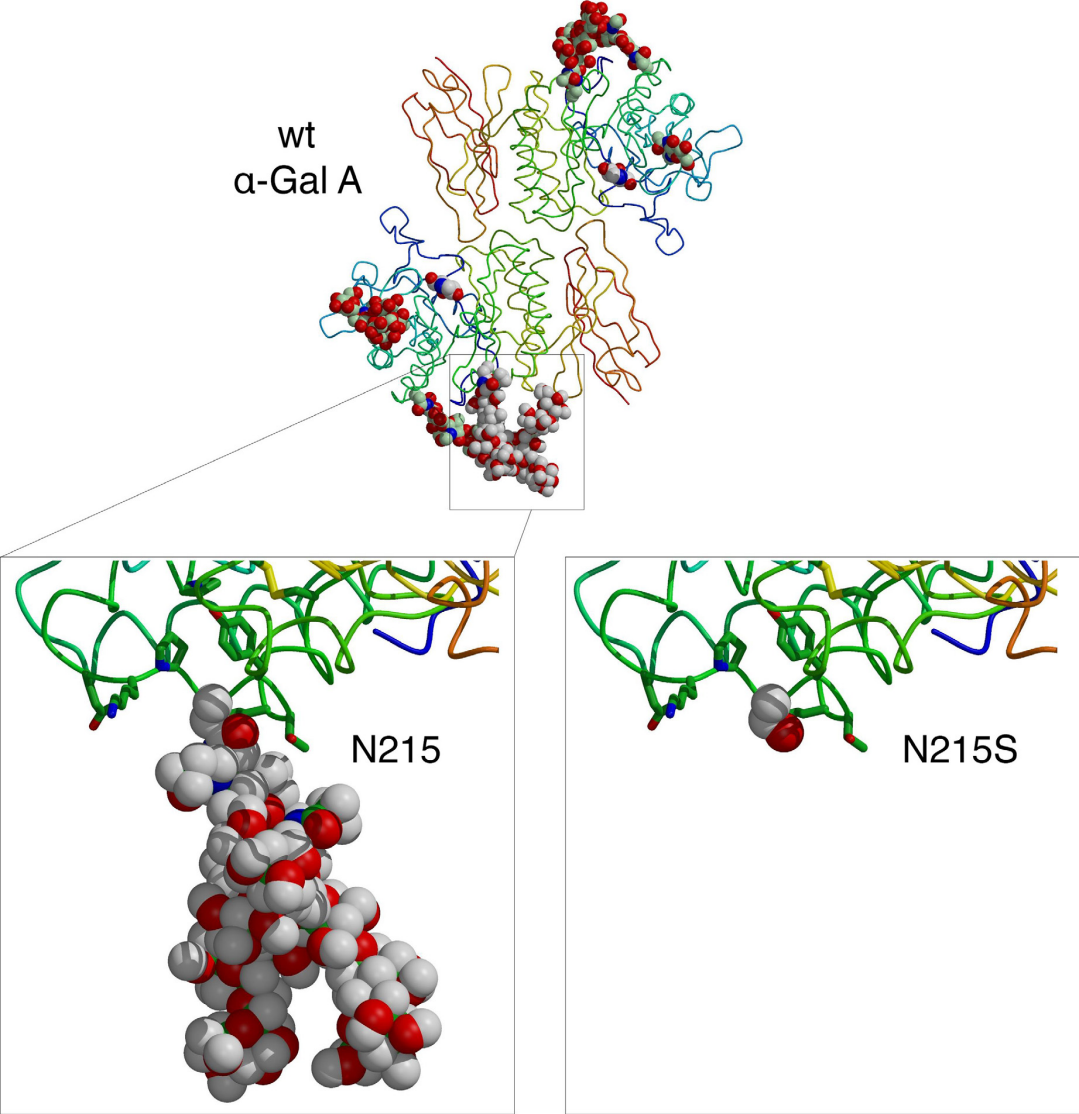
The overall structure of wild‐type α‐Gal is shown at the top, with a zoom of the N215 carbohydrate region at lower left. At lower right, the p.N215S mutation has been computationally modeled from the wild‐type (wt) structure. The serine at 215 is easily accommodated into the structure, but the resulting loss of the large N‐linked glycan could affect the folding and/or trafficking of the p.N215S mutant α‐Gal enzyme

The substitution of a serine for an asparagine at codon 215 of α‐Gal can have major implications. When wild‐type α‐Gal enzyme is expressed in Chinese hamster ovary or human cells, the N‐linked glycan attached at N215 is either high mannose, hybrid or complex (Lee et al., 2003). This glycan can be used for the trafficking of α‐Gal to the lysosome. The p.N215S mutation lacks the (large) N‐linked glycan attachment site at 215 (Figure 4). These N‐linked glycans can help stabilize glycoprotein structures through protein–glycan interactions and have critical roles in the folding and trafficking of macromolecules (Hebert, Lamriben, Powers, & Kelly, 2014). However, the crystal structure of α‐Gal shows that the N215 carbohydrate extends away from the surface of the protein. As seen in Figure 4, the smaller serine can be accommodated easily by the structure of α‐Gal. Thus, the loss of the glycan at 215 could lead to loss of interactions with macromolecular chaperones such as calnexin and calreticulin. Additionally, because the N‐linked glycan helps the α‐Gal molecule traffic to the lysosome, loss of the glycan at 215 may affect the subcellular distribution of the enzyme. All in all, the p.N215S mutation appears to have altered folding and trafficking of α‐Gal to the lysosome. However, the reason why patients harboring the p.N215S mutation show almost no renal involvement remains to be ascertained.

This study has some limitations. Our analysis included patients’ first renal and cardiac assessments during natural history follow‐up, as recorded in the Fabry Registry. The start of natural history follow‐up varies for individual patients and is dependent on the age at definitive diagnosis of Fabry disease, which in turn depends on the severity of symptoms and the method used to establish diagnosis (clinical suspicion, family screening, high‐risk‐population screening, neonatal screening). Data may not have been entered retrospectively for enrolled patients, and given that newly diagnosed severe classic patients may be initiated on ERT soon after diagnosis without all recommended assessments having been performed, natural history data may be incomplete. This also applies to documentation of early disease symptoms in p.N215S patients diagnosed well into adulthood, which may not be fully available in the Fabry Registry. Furthermore, classic patients are more likely to be initiated on ERT and at a younger age (particularly male patients). Therefore, the rate of severe clinical events during natural history follow‐up should be interpreted with caution. Cardiac or renal biopsy data from p.N215S patients were not available in the Fabry Registry. Cardiovascular risk factors including the extent of myocardial fibrosis, hypertension, hypercholesterolemia, obesity, tobacco use, lack of physical activity, and diabetes have not been assessed in this analysis. Although unlikely, Fabry mutations currently categorized as classic in the Fabry‐database.org mutation database may eventually prove to be associated with a later‐onset phenotype based on evolving knowledge about the mutation's phenotypic characteristics. Incomplete data sets for left ventricular mass (indexed), urine protein‐to‐creatinine ratio, Fabry disease biomarkers [e.g. globotriaosylceramide (GL‐3), globotriaosylsphingosine (lyso‐GL‐3)], and chronic white matter hyperintensities on brain magnetic resonance imaging precluded meaningful analysis of these parameters. Data from patients were analyzed per age category and, therefore, these analyses are cross‐sectional rather than longitudinal. Not all p.N215S Fabry disease patients have been diagnosed and enrolled in the Fabry Registry. This may be particularly applicable to female heterozygotes. Relatively few females were included in this analysis, since, given the X‐linked inheritance pattern, the female‐to‐male ratio should approach 2:1 (Germain & Jurca‐Simina, 2018). A patient selection bias toward the inclusion of more severely affected patients cannot be excluded, whereas asymptomatic patients may not have come to medical attention. The Fabry Registry contains observational data of Fabry patients only. It is not a prospective clinical trial, and data interpretation is limited by the lack of an appropriately matched control group.

## 5 CONCLUSIONS

This study analyzed the clinical characteristics of 59 male and 66 female Fabry patients with the p.N215S mutation and represents the largest sample size with this type of data reported to date. The data confirm that p.N215S is a disease‐causing Fabry mutation. Severe clinical manifestations are found primarily in the heart until late adulthood and, particularly in male patients, cardiac involvement appears to be progressive and may become as severe as (or occasionally more severe than) in patients with classic Fabry disease. Renal impairment was observed in few male p.N215S patients later in life and was uncommon in females. If observed, monitoring of renal function (GFR and albuminuria/proteinuria) and exclusion of other Fabry‐unrelated causes of renal insufficiency are warranted. Once abnormalities have been observed in patients with the Fabry p.N215S genotype, early diagnosis and close monitoring of cardiac status is important because Fabry‐specific therapeutic intervention and treatment of cardiovascular risk factors may be indicated, as well as cascade family screening for early diagnosis of family members.

## CONFLICT OF INTEREST

Germain: Fabry Registry Board member; honoraria from Amicus Therapeutics, Sanofi Genzyme and Shire HGT; consulting fees from Sanofi Genzyme and Shire HGT. Brand: member of the German Association of the Scientific Medical Societies; institutional honoraria and research grants from Amicus Therapeutics, Sanofi Genzyme and Shire HGT. Burlina: honoraria for presentations and board meetings from Amicus Therapeutics, Merck‐Serono, Nutricia International and Sanofi Genzyme; Fabry Registry Board member and Nutricia International Advisory Board member. Cecchi: honoraria for lectures and research grants from Sanofi Genzyme. Garman: consulting fees and honoraria for Fabry disease lectures from Amicus Therapeutics and Sanofi Genzyme. Kempf: former employee of Sanofi. Laney: Fabry Registry Board member; consulting fees from Sanofi Genzyme; investigator/coordinator in clinical trials sponsored by Alexion, Amicus Therapeutics, Pfizer, Protalix BioTherapeutics, Retrophin, Sanofi Genzyme, Shire HGT and Synageva (activities found to be in compliance with conflict of interest policies at Emory University). Linhart: Fabry Registry Board member; honoraria and consulting fees from Amicus Therapeutics, Sanofi Genzyme and Shire HGT. Maródi: research funds and travel support from Sanofi Genzyme. Nicholls: investigator in clinical trials sponsored by Amicus Therapeutics, Protalix BioTherapeutics, Sanofi Genzyme and Shire HGT; travel support and participated in Fabry Disease Advisory Boards for Amicus Therapeutics, Sanofi Genzyme and Shire HGT. Ortiz: Fabry Registry Board member; honoraria from Sanofi Genzyme and Shire HGT; consulting fees from Sanofi Genzyme. Pieruzzi: Advisory Board fees and honoraria for lectures from Amicus Therapeutics, Sanofi Genzyme and Shire HGT. Shankar: primary investigator in clinical trials and received research support and educational grants sponsored by Actelion, Amicus Therapeutics, Protalix BioTherapeutics, Sanofi Genzyme and Shire HGT; honoraria and travel support as speaker and for investigator meetings of Protalix BioTherapeutics, Sanofi Genzyme and Shire HGT (activities found to be in compliance with conflict of interest policies at Emory University). Waldek: former Fabry Registry Board member; honoraria from Amicus Therapeutics, Sanofi Genzyme and Shire HGT; consulting fees from Sanofi Genzyme and Shire HGT. Wanner: Fabry Registry Board member; honoraria from Sanofi Genzyme and a institutional research grant. Jovanovic: Fabry Registry Board member; Advisory Board fees and honoraria for Fabry disease lectures from Amicus Therapeutics, Sanofi Genzyme and Shire HGT.
