# Supplementary material for: Phenotypic characteristics of the p.Asn215Ser (p.N215S) GLA mutation in male and female patients with Fabry disease: A multicenter Fabry Registry study
Source: Mol Genet Genomic Med. 2018 Apr 12;6(4):492–503. doi: 10.1002/mgg3.389 (PMC6081232; doi:10.1002/mgg3.389)
Supplement: Supplementary file 1 [file MGG3-6-492-s001.docx]

Table S1. Mutations associated with classic Fabry disease reported for 401 Fabry Registry patients from the clinical centers participating in the study

| **Mutation** | **Patients, n** |  | **Mutation** | **Patients, n** |  | **Mutation** | **Patients, n** |
| --- | --- | --- | --- | --- | --- | --- | --- |
| p.A31V | 7 |  | p.G183D | 4 |  | p.Q330X | 2 |
| p.P40S | 3 |  | p.G183S | 5 |  | p.W340X | 2 |
| p.M42V | 1 |  | p.M187T | 1 |  | p.R342Q | 26 |
| p.G43V | 1 |  | p.M187V | 3 |  | p.R342X | 14 |
| p.W44X | 2 |  | p.V199M | 1 |  | p.W349X | 5 |
| p.H46R | 2 |  | p.C202W | 6 |  | p.N355K | 2 |
| p.H46Y | 1 |  | p.C202Y | 2 |  | p.R356W | 3 |
| p.R49G | 1 |  | p.W204X | 3 |  | p.Q357X | 2 |
| p.C52R | 13 |  | p.P205T | 6 |  | p.E358G | 3 |
| p.C52S | 1 |  | p.R220X | 15 |  | p.E358K | 2 |
| p.C56X | 2 |  | p.Q221X | 1 |  | p.G361R | 1 |
| p.M72I | 1 |  | p.C223Y | 1 |  | p.P362L | 4 |
| p.S78X | 2 |  | p.N224D | 1 |  | p.R363C | 1 |
| p.E79X | 1 |  | p.W226X | 2 |  | p.G373S | 5 |
| p.D92Y | 1 |  | p.R227Q | 13 |  | p.W399X | 3 |
| p.D93N | 3 |  | p.R227X | 14 |  | p.P409A | 2 |
| p.C94S | 6 |  | p.W236C | 3 |  | c.124_125del | 1 |
| p.C94Y | 3 |  | p.W236R | 2 |  | c.154del | 1 |
| p.R100K | 2 |  | p.W236X | 1 |  | c.318_328del | 1 |
| p.R100T | 1 |  | p.L243F | 6 |  | c.369+1G>A | 4 |
| p.R112C | 28 |  | p.W245X | 2 |  | c.547+1G>A | 1 |
| p.Q119X | 3 |  | p.Q250X | 2 |  | c.548-1G>A | 3 |
| p.A121P | 6 |  | p.W262X | 12 |  | c.639+1G>A | 1 |
| p.A135V | 11 |  | p.D266E | 1 |  | c.718_719del | 8 |
| p.T141I | 1 |  | p.D266V | 3 |  | c.801+2T>C | 1 |
| p.A143P | 3 |  | p.W277X | 2 |  | c.801+3A>G | 8 |
| p.Y151X | 2 |  | p.W287C | 3 |  | c.1000-1G>C | 1 |
| p.D155H | 2 |  | p.P293T | 2 |  | c.1011_1029del | 1 |
| p.A156D | 1 |  | p.L294X | 2 |  | c.1030_1031del | 2 |
| p.Q157X | 5 |  | p.N298S | 1 |  | c.1033_1034del | 2 |
| p.W162C | 4 |  | p.R301P | 8 |  | c.1074_1075del | 1 |
| p.L167Q | 1 |  | p.R301X | 12 |  | c.1207del | 1 |
| p.G171R | 3 |  | p.I303N | 2 |  | c.1223del | 2 |
| p.C172G | 1 |  | p.Q306X | 1 |  | c.1235_1236del | 1 |
| p.C172Y | 3 |  | p.Q327K | 5 |  | c.1277_1278del | 1 |
| p.Y173X | 3 |  | p.G328R | 4 |  |  |  |

Sequence of reference: NM_000169.2.
